# Population-Based Digital Health Interventions to Deliver at-Home COVID-19 Testing: SCALE-UP II Randomized Clinical Trial

**DOI:** 10.2196/74145

**Published:** 2025-07-28

**Authors:** Guilherme Del Fiol, Tatyana V Kuzmenko, Brian Orleans, Jonathan J Chipman, Tom Greene, Ray Meads, Kimberly A Kaphingst, Bryan Gibson, Kensaku Kawamoto, Andy J King, Tracey Siaperas, Shlisa Hughes, Alan Pruhs, Courtney Pariera Dinkins, Cho Y Lam, Joni H Pierce, Ryzen Benson, Emerson P Borsato, Ryan C Cornia, Leticia Stevens, Richard L Bradshaw, Chelsey R Schlechter, David W Wetter

**Affiliations:** 1 Department of Biomedical Informatics, University of Utah, 421 Wakara Way, Suite 140, Salt Lake City, UT, 84108, United States, 1 8015814080; 2 Huntsman Cancer Institute, University of Utah, Salt Lake City, UT, USA; 3 Department of Population Health Sciences, University of Utah, Salt Lake City, UT, United States; 4 Department of Communication, University of Utah, Salt Lake City, UT, USA; 5 Association for Utah Community Health, Salt Lake City, UT, United States; 6 University of California, San Francisco, CA, USA

**Keywords:** digital health, chatbots, text messaging, COVID-19, at-home testing

## Abstract

**Background:**

Digital health interventions could be a scalable approach to delivering at-home COVID-19 testing.

**Objective:**

SCALE-UP II aimed to investigate the effectiveness of 3 digital health interventions on the delivery of mailed at-home COVID-19 testing: SMS text messaging, automated chatbot, and patient navigation upon request.

**Methods:**

The study was a pragmatic randomized controlled trial. Participants who self-reported that they had a smartphone were randomized in a 2×2×2 factorial design (smartphone study) to receive (1) chatbot or text messaging, (2) the option to request patient navigation, and (3) intervention frequency every 10 or 30 days. All other participants were randomized in a 2×2 factorial design (nonsmartphone study) to receive the option to request patient navigation and intervention frequency every 10 or 30 days. Study settings were safety net community health centers located across the state of Utah, United States. Eligible patients were >18 years old, with a primary care visit in the last 3 years, and a valid cellphone in the community health centers electronic health record. The primary outcome was the proportion of participants requesting at-home COVID-19 tests.

**Results:**

The trial enrolled 2117 in the smartphone study and 31,439 in the nonsmartphone study. In the smartphone study, the proportion of participants who requested test kits in the Chatbot arm was lower than in SMS text messaging (174/1051, 16.6% vs 555/1066, 52.1%; adjusted risk ratio (aRR) 0.317, 98.33% CI 0.27‐0.38; *P*<.001). In the nonsmartphone study, the proportion of participants who requested test kits was higher if they were messaged every 10 days rather than every 30 days (860/15,717, 5.5% vs 752/15,722, 4.8%; aRR 1.144, 97.5% CI 1.03‐1.28; *P*=.005). However, participants in the 10-day versus 30-day condition were more likely to opt out of receiving study interventions (1977/15,717, 12.6% vs 1147/15,722, 7.3%; aRR 1.72, 97.5% CI 1.59‐1.86; *P*<.001). In the nonsmartphone study, the proportion of participants who requested test kits was lower for those in the patient navigation condition compared with no patient navigation (680/15,718, 4.3% vs 932/15,721, 5.9%; aRR 0.729, 97.5% CI 0.65‐0.81; *P*<.001).

**Conclusions:**

Simple bidirectional text messaging was more effective than an interactive web-based chatbot on the delivery of COVID-19 testing. Although messaging every 10 days was more effective than every 30 days, it also led to a larger opt-out rate. Digital health interventions based on automated bidirectional SMS text messaging are a simple, scalable, and low-cost strategy to offer access to at-home COVID-19 testing. Similar approaches may be used to support public health response and other forms of at-home testing.

## Introduction

Individuals from populations, such as racial and ethnic minorities, low socioeconomic status (SES), and those from rural areas, experienced profound health disparities in the COVID-19 pandemic, including lower vaccination rates, higher hospitalization rates, and higher mortality [1-8]. Timely testing is crucial to reduce exposure and provide timely treatment to those at higher risk for severe disease. While at-home tests provide a convenient, rapid, and relatively low-cost option for self-testing [9,10], significant disparities persist in the uptake of at-home testing. For example, the use of at-home testing increased 5-fold from 2% of individuals surveyed in October 2021 to 11% in January 2022; however, in the United States, self-tests were over twice as likely to be used by individuals identifying as White, those with high SES, and those holding postgraduate degrees [11]. Thus, scalable approaches are needed to help reduce disparities in the uptake of at-home testing for conditions such as COVID-19 infection.

Population-level interventions using digital health tools, such as SMS text messaging and chatbots, and coupled with a human component, are a promising approach for promoting equitable access to preventive care services. Despite disparities in access to digital technologies, cellphone ownership is now ubiquitous. Even in US households earning less than US $30,000 annually, 97% have a cellphone and 76% have a smartphone [12]. Meta-analyses have found that population health interventions using SMS text messaging are effective in improving compliance with preventive care, beneficial for multiple demographic groups, and inexpensive to deliver [13-17]. Chatbots are automated interactive agents designed to mimic human interaction. They have many advantages for patient engagement, including providing scripted education interactively, chunking information into small segments that are easier to process, and allowing for choice in the amount of information received. Chatbots have been shown to be effective across a wide variety of health domains, including asthma, diabetes, pain management, physical activity, mental health, and genetic testing [18-24]. Finally, digital health interventions can also be coupled with a human component. For example, the Community Preventive Services Task Force (CPSTF) recommended the use of patient navigation (PN) for increasing breast, cervical, and colorectal cancer screening to reduce health inequities [25].

The SCALE-UP II pragmatic trial used a factorial design to examine the effect of 3 patient health management interventions, SMS text messaging, chatbot, and “reactive” (upon patient request) PN, on reach and uptake of at-home COVID-19 testing among patients receiving care at safety net community health centers (CHCs) located in urban and rural areas across the state of Utah. The trial aimed to compare the proportion of patients requesting a mailed COVID-19 test kit when randomized to (1) text messaging versus chatbot, (2) interventions every 10 days versus 30 days, and (3) no access to PN versus access to reactive PN.

## Methods

### Trial Design

**Figure 1.**
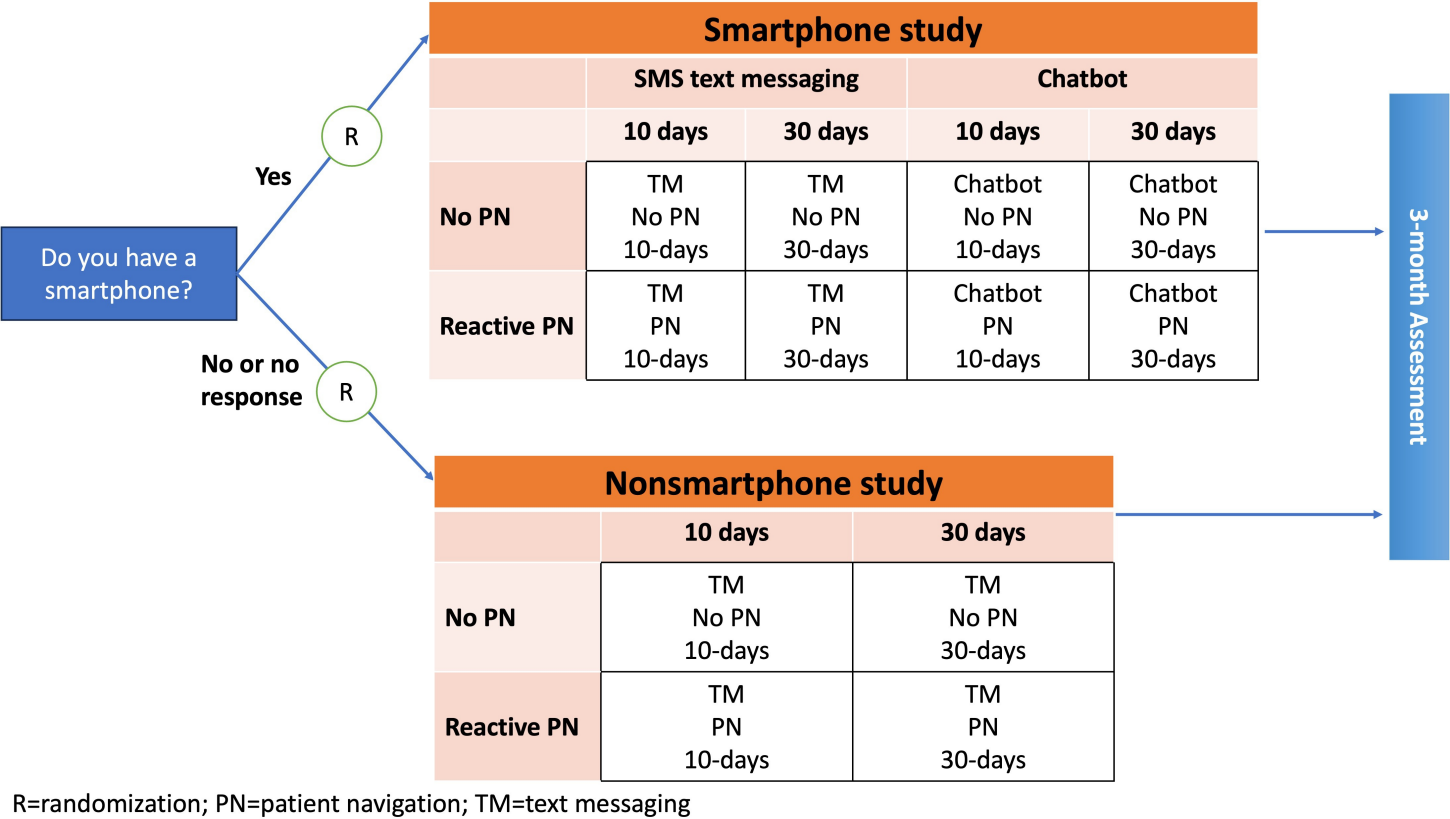
SCALE-UP II trial design with randomization into the smartphone and nonsmartphone studies based on participants’ responses to an introductory message asking about smartphone ownership. PN: patient navigation; TM: text messaging.

SCALE-UP II was a multisite, pragmatic clinical trial with patients randomized at the individual level in one of 2 study designs based on patients’ response to a SMS text message asking if the patient owned a smartphone, which was required for accessing the chatbot (Figure 1) [26]. Those who self-reported that they owned a smartphone were randomized in a 2×2×2 factorial design (smartphone study) to receive (1) chatbot or text messaging, (2) reactive (ie, available upon participant request) PN (yes or no); and (3) intervention frequency every 10 or 30 days (total of 6 or 3 messages, respectively). All other participants, that is, those who did not respond to the initial SMS text message or replied indicating they did not have a smartphone, were randomized in a 2×2 factorial design (nonsmartphone study) to receive text messaging (1) with or without reactive patient navigation and (2) intervention frequency every 10 or 30 days (total of 6 or 3 messages, respectively) [27]. We considered enrolling all participants into a single study with a chatbot condition. However, we were concerned that a sizable proportion of participants (~25%) would not have a smartphone with internet access and therefore would be unable to access the chatbot and benefit from the intervention. Therefore, we opted for a more equitable design that ensured maximal reach.

### Settings and Study Population

SCALE-UP II was conducted in partnership with the Association for Utah Community Health (AUCH) and its affiliated CHCs, and incorporated a multipronged community engagement approach [28]. Three CHCs with 12 primary care clinics affiliated with AUCH volunteered for participation. These CHCs serve over 39,000 patients per year, with 18,057 (18,057/39,000, 46.3%) Hispanic or Latino, 21,294 (21,294/39,000, 54.6%) uninsured, and 3 of the 12 clinics located in rural areas. Eligible patients (1) had an appointment at one of the participating CHCs in the last 3 years, (2) were 18 years and older, and (3) had a valid cellphone number recorded in the CHC electronic health record (EHR). Patients were excluded if they (1) opted out from the previous SCALE-UP I trial [27], (2) were enrolled in a concurrent tobacco cessation trial [29], or (3) had a duplicate phone number with another patient (in this case, only the patient with the most recent visit was included).

### Interventions

All study interventions (1) were sent on behalf of the participant’s clinic, (2) included an option to request free at-home COVID-19 test kits mailed to the patient for use as needed, (3) asked participants to confirm the accuracy of their address recorded in the EHR with the option to correct their address for mailing of test kits, (4) were provided in English or Spanish based on the patient’s preferred language recorded in the EHR, and (5) allowed participants to reply “STOP” to opt-out at any time (Figures S1 and S2 in Multimedia Appendix 1 and verbatim SMS text messaging scripts in the Multimedia Appendix 1).

#### Text Messaging

Text messaging consisted of simple bidirectional SMS text messaging with the option to reply “YES” to request at-home COVID-19 test kits (Figure S1 in Multimedia Appendix 1, left).

#### Chatbot

Participants randomized to the chatbot intervention received an SMS text message with a hyperlink (Figure S1 in Multimedia Appendix 1, right) to access the chatbot on their phone’s web browser. The chatbot used a predefined script (ie, a fixed set of questions and scripted answers) with a list of topics addressing knowledge gaps and hesitancy factors related to at-home COVID-19 testing (Figure S2 in Multimedia Appendix 1), such as benefits of testing, when to test, test accuracy, how to use a test, and guidance based on the test result. At any point in the chat, participants could click “Yes, send me a test” to request a test kit. The script was created through an iterative user-centered design, with input from experts in health communication, public health, and health behaviors, as well as from our Study Advisory Committee and Patient Advisory Committee members [26].

#### Patient Navigation

SCALE-UP II used community health workers used by AUCH as patient navigators to address practical barriers (eg, access to treatment in case of a positive test), motivation, and hesitancy to COVID-19 testing. Patient navigation in SCALE-UP II was “reactive” such that participants had to explicitly request to speak to a patient navigator (Multimedia Appendix 1, eFigure 1).

### Measures

#### Primary Outcomes and Hypotheses

The primary outcome per the study protocol was testing: the proportion of trial participants using an at-home COVID-19 test at a 90-day follow-up. However, due to a very low response rate in self-reported use of at-home testing (1306/2117, 61.7% in the nonsmartphone study and 12,800/31,439, 40.7% in the smartphone study), we decided to change the primary outcome to Reach-Accept Testing, that is, the proportion of participants who requested a test kit and confirmed their mailing address. This decision was made before examination of the effects of the interventions on outcomes as consensus between the principal investigators and the statistical team for the following reasons. Testing was self-reported, and the very low response rates were associated with intervention arm and plausibly with the outcome. Study interventions offered participants the opportunity to opt out anytime during the study, and those who opted out were not contacted for self-report testing. Significant differences in opt-out rates were found between study conditions (Tables1  2), leading to systematic differences in follow-up rates for the testing outcome. The analysis reported in the Multimedia Appendix file (Tables S8 and S9 in Multimedia Appendix 1) provides strong evidence that response rates among those who received the survey varied by study condition. For a valid comparison, and given the extent of missing outcomes, multiple imputations would need to rely on very strong information missing at random assumptions, that is, the imputation covariates account for why outcomes were missing and the outcomes themselves. In contrast, Reach-Accept testing was automatically collected as a part of the study interventions, so that there were no missing data observed. We could reliably perform an intention to treat analysis. Finally, Reach-Accept testing was in the same pathway as testing in that requesting a test was the immediate step preceding the use of a test and having test kits readily available at home for use whenever needed is still an important public health outcome.

**Table 1.** Primary and secondary outcomes for the smartphone study

Study condition	Total number (N)	Reach-Accept testing			Reach-Engage			Opt-out		
		n (%)	aRR^a^ (98.3% CI)	*P* value	n (%)	aRR (98.3% CI)	*P* value	n (%)	aRR (98.3% CI)	*P* value
Delivery mechanism										
TM^b^ [Ref]	1066	555 (52.1)	—^c^	—	625 (58.6)	—	—	50 (4.7)	—	—
Chatbot	1051	174 (16.6)	0.317 (0.27-0.38)	<.001	309 (29.4)	0.502 (0.44-0.57)	<.001	56 (5.3)	1.172 (0.75-1.83)	.39
Outreach frequency										
Every 30 days [Ref]	1057	348 (32.9)	—	—	440 (41.6)	—	—	27 (2.6)	—	—
Every 10 days	1060	381 (35.9)	1.088 (0.96-1.24)	.12	494 (46.6)	1.099 (0.99-1.22)	.04	79 (7.5)	2.857 (1.70-4.80)	<.001
PN^d^										
PN not offered [Ref]	1055	369 (35.0)	—	—	472 (44.7)	—	—	42 (4.0)	—	—
PN offered	1062	360 (33.9)	0.976 (0.86-1.11)	.65	462 (43.5)	0.998 (0.90-1.11)	.96	64 (6.0)	1.463 (0.93-2.30)	.04

a aRR: adjusted risk ratio.

b TM: text messaging.

c Not available.

d PN: patient navigation.

**Table 2.** Primary and secondary outcomes for the nonsmartphone study.

Study condition	Total number (N)	Reach-Accept testing			Reach-Engage			Opt-out		
		n (%)	aRR^a^ (97.5% CI)	*P* value	n (%)	aRR (97.5% CI)	*P* value	n (%)	aRR (97.5% CI)	*P* value
Outreach frequency										
Every 30 days [Ref]	15,722	752 (4.8)	—^b^	—	1191 (7.6)	—	—	1147 (7.3)	—	—
Every 10 days	15,717	860 (5.5)	1.144 (1.03-1.28)	.01	1388 (8.8)	1.166 (1.07-1.27)	<.001	1977 (12.6)	1.718 (1.59-1.86)	<.001
PN^c^										
PN not offered [Ref]	15,721	932 (5.9)	—	—	1422 (9.0)	—	—	1565 (10.0)	—	—
PN offered	15,718	680 (4.3)	0.729 (0.65-0.81)	<.001	1157 (7.4)	0.813 (0.75-0.89)	<.001	1559 (9.9)	0.996 (0.92-1.07)	.9

a aRR: adjusted risk ratio.

b Not available.

c PN: patient navigation.

The primary hypotheses for the smartphone study were main effects for delivery mechanism (ie, Chatbot more effective than SMS text messaging), main effects for PN (ie, PN more effective than no PN), and main effects for message frequency (ie, exposure to messages every 10 d more effective than every 30 d). For the nonsmartphone study, the primary hypotheses were main effects for PN and main effects for message frequency. These hypotheses were adjusted for multiple comparisons using the Bonferroni method at an alpha level of .0167 for the smartphone study to account for 3 coprimary comparisons, and .0250 for the nonsmartphone study to account for 2 coprimary comparisons.

#### Secondary Analyses and Implementation Outcomes

Testing was analyzed as a secondary outcome. Implementation outcomes included Reach-Engage (proportion of participants in SMS text messaging who sent any reply other than opt-out to the SMS text message offering a test kit, or proportion in chatbot who clicked on the hyperlink to launch the chatbot), PN-Request (proportion in PN who requested PN), PN-Engage (proportion in PN who talked to a patient navigator), and Opt-Out (proportion who replied any time in the study requesting to stop receiving study interventions).

#### Study Assessments

The primary outcome Reach-Accept Testing, and secondary outcomes Reach-Engage, PN-Request, PN-Engage, and Opt-Out were obtained from computer system logs. Patient demographics (eg, age, sex, race, ethnicity, rural vs urban, and language) were extracted from CHC EHR data. Testing was collected through 2 methods from patients who requested a test kit: (1) a brief SMS text message sent 90 days after the first exposure to interventions asking if they used the COVID-19 test at least once, and (2) a survey sent 5-8 days after the brief SMS text message. We used the testing outcome reported via SMS text message if available, and the testing outcome from the survey otherwise. Investigators were blinded to participant allocation and outcomes. As described above, the study received a waiver of consent such that participants were blinded to trial enrollment and participation. Study outcomes were collected through automated methods as a part of the digital health interventions; therefore, outcome assessment was also blinded.

### Statistical Analysis

Separate analyses were conducted for the smartphone study and the nonsmartphone study. Log-binomial models were used to regress Reach-Accept Testing upon each of the 3 main effects in the smartphone study, that is, chatbot (vs SMS text messaging), patient navigation (vs no PN), and outreach frequency (10 vs 30 d); and 2 main effects in the nonsmartphone study, that is, PN (vs no PN) and outreach frequency (10 vs 30 d). The log-binomial models included covariates to adjust for randomization stratification variables (rural vs urban, CHC, and randomization arm in our previous SCALE-UP I trial [27]) and a categorical variable reflecting the calendar month when the participant received the first intervention message. Adjusted risk ratios (aRR) and CIs were reported for each main effect. In preliminary analyses reported in the Multimedia Appendix 1, the pairwise and 3-way interactions between the main effects were added to the log-binomial models described above in order to assess for any effect modifications across interventions, but these analyses did not support the inclusion of the interactions.

### Ethical Considerations

This study was approved by the University of Utah Institutional Review Board (protocol 00150669) and follows the CONSORT (Consolidated Standards of Reporting Trials) reporting guidelines. The trial was registered with Clinicaltrials.gov (NCT05533359 for the Smartphone study and NCT05533918 for the Nonsmartphone study). As a pragmatic trial with minimal risk, the University of Utah Institutional Review Board approved a waiver of consent for enrollment. Therefore, all eligible participants were automatically enrolled in and blinded to the study.

## Results

### Participant Characteristics

**Figure 2.**
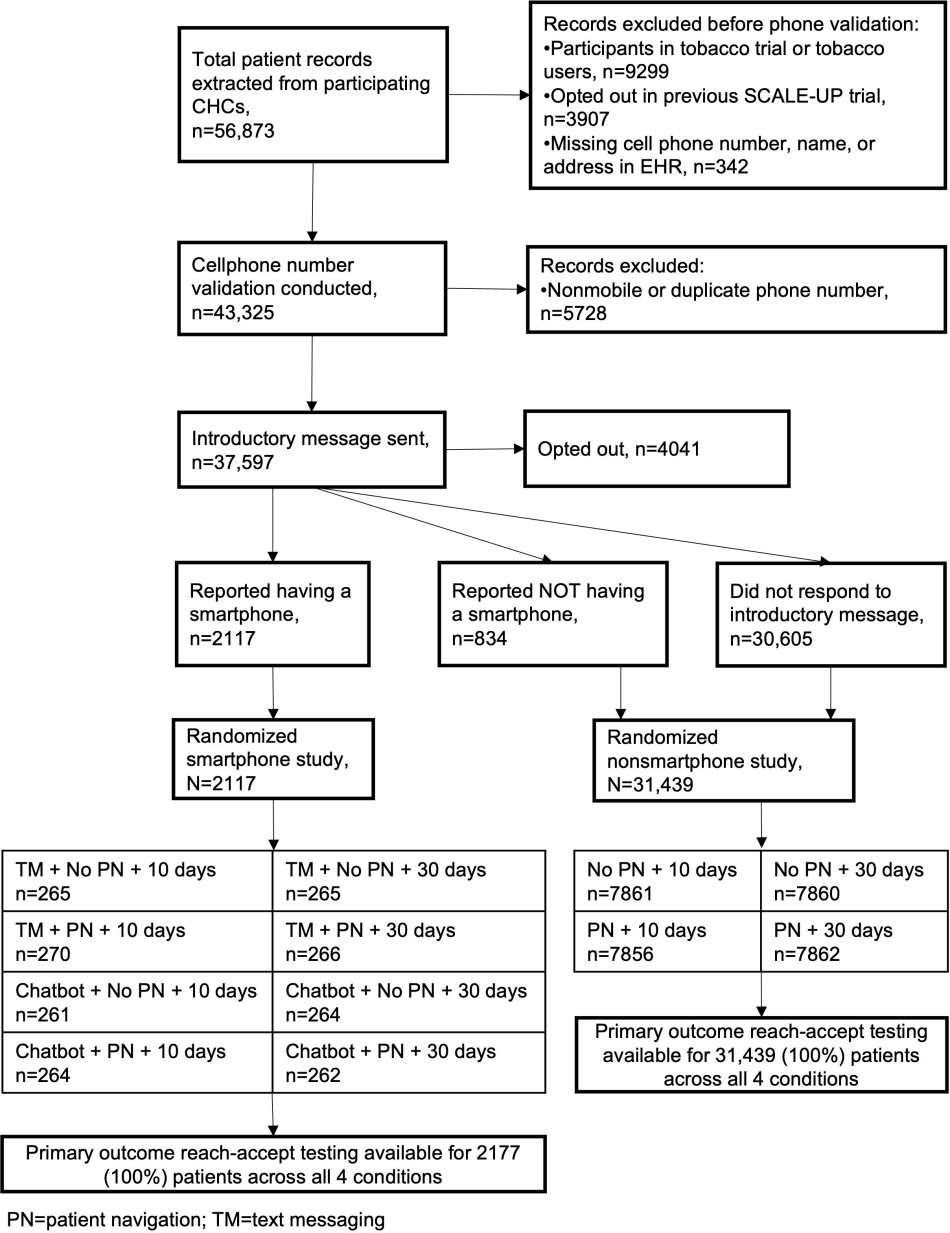
Trial flow. CHC: community health center; EHR: electronic health record; PN: patient navigation; TM: text messaging.

Both studies took place from December 19, 2022, to August 28, 2023. Of 56,873 adult patients with a primary care appointment in the last 3 years, 19,276 (33.9%) were excluded and 4041 (10.8%) opted out upon receiving the introductory message, yielding 33,556 (59%) eligible patients for both studies (Figure 2). Out of those patients, 2951 (8.8%) replied to the introductory message, with 2117 (6.3%) reporting that they had a smartphone and were randomized to the smartphone study. The remaining 31,439 (93.7%) participants were randomized to the nonsmartphone study.

The mean age of participants at initial study launch was 42.6 (SD 16.2) years. Overall, the majority of patients in the studies were female (19,235/33,556, 57.3%), Latino (18,232/33,556, 54.3%), lived in an urban area (30,216/33,556, 90%), had a preference for a language other than English recorded in the EHR (16,238/33,556, 51.6%), and were uninsured (20,546/33,556, 61.2%; Table 3).

**Table 3.** Overall patient population characteristics by experimental condition (N=33,556).

Participant characteristics	Both studies	Smartphone study							Nonsmartphone study				
		Overall	Patient navigation		Outreach frequency		Delivery mechanism		Overall	Patient navigation		Outreach frequency	
			No PN^a^	PN	30 days	10 days	TM^b^	Chatbot		No PN	PN	30 days	10 days
Total number (N)	33,556	2117	1055	1062	1057	1060	1066	1051	31,439	15,721	15,718	15,722	15,717
Age (years), mean (SD)	42.6 (16.2)	49.0 (14.6)	49.0 (14.64)	49.0 (14.6)	48.8 (14.5)	49.2 (14.8)	48.9 (14.5)	49.1 (14.8)	42.2 (16.2)	42.2 (16.2)	42.2 (16.2)	42.1 (16.1)	42.3 (16.2)
Sex, n (%)													
Female	19,235 (57.3)	1451 (68.5)	715 (67.8)	736 (69.3)	716 (67.7)	735 (69.3)	740 (69.4)	711 (67.6)	17,784 (56.6)	8908 (56.7)	8876 (56.5)	8873 (56.4)	8911 (56.7)
Male	14,318 (42.7)	665 (31.4)	340 (32.2)	325 (30.6)	341 (32.3)	324 (30.6)	326 (30.6)	339 (32.3)	13,653 (43.4)	6812 (43.3)	6841 (43.5)	6848 (43.6)	6805 (43.3)
Unknown	3 (0.0)	1 (0.0)	0 (0.0)	1 (0.1)	0 (0.0)	1 (0.1)	0 (0.0)	1 (0.1)	2 (0.0)	1 (0.0)	1 (0.0)	1 (0.0)	1 (0.0)
Race or Ethnicity, n (%)													
American Indian or Alaska Native	80 (0.2)	3 (0.1)	1 (0.1)	2 (0.2)	3 (0.3)	0 (0.0)	1 (0.1)	2 (0.2)	77 (0.2)	45 (0.3)	32 (0.2)	35 (0.2)	42 (0.3)
Asian	331 (1.0)	14 (0.7)	6 (0.6)	8 (0.8)	8 (0.8)	6 (0.6)	9 (0.8)	5 (0.5)	317 (1.0)	160 (1.0)	157 (1.0)	167 (1.1)	150 (1.0)
Black	413 (1.2)	19 (0.9)	10 (0.9)	9 (0.8)	9 (0.9)	10 (0.9)	9 (0.8)	10 (1.0)	394 (1.3)	185 (1.2)	209 (1.3)	190 (1.2)	204 (1.3)
Hawaiian or Pacific Islander	213 (0.6)	8 (0.4)	4 (0.4)	4 (0.4)	3 (0.3)	5 (0.5)	4 (0.4)	4 (0.4)	205 (0.7)	102 (0.6)	103 (0.7)	106 (0.7)	99 (0.6)
Hispanic or Latino	18,232 (54.3)	1158 (54.7)	578 (54.8)	580 (54.6)	585 (55.3)	573 (54.1)	586 (55.0)	572 (54.4)	17,074 (54.3)	8468 (53.9)	8606 (54.8)	8533 (54.3)	8541 (54.3)
Multirace	340 (1.0)	25 (1.2)	12 (1.1)	13 (1.2)	13 (1.2)	12 (1.1)	9 (0.8)	16 (1.5)	315 (1.0)	160 (1.0)	155 (1.0)	161 (1.0)	154 (1.0)
White	9977 (29.7)	719 (34.0)	360 (34.1)	359 (33.8)	352 (33.3)	367 (34.6)	364 (34.1)	355 (33.8)	9258 (29.4)	4648 (29.6)	4610 (29.3)	4615 (29.4)	4643 (29.5)
Unknown	3970 (11.8)	171 (8.1)	84 (8.0)	87 (8.2)	84 (7.9)	87 (8.2)	84 (7.9)	87 (8.3)	3799 (12.1)	1953 (12.4)	1846 (11.7)	1915 (12.2)	1884 (12.0)
Urban vs rural (urban), n (%)	30,216 (90.0)	1840 (86.9)	916 (86.8)	924 (87.0)	921 (87.1)	919 (86.7)	922 (86.5)	918 (87.3)	28,376 (90.3)	14,189 (90.3)	14,187 (90.3)	14,188 (90.2)	14,188 (90.3)
Language, n (%)													
English	16,238 (48.4)	1026 (48.5)	511 (48.4)	515 (48.5)	520 (49.2)	506 (47.7)	505 (47.4)	521 (49.6)	15,212 (48.4)	7632 (48.5)	7580 (48.2)	7570 (48.1)	7642 (48.6)
Spanish	14,698 (43.8)	993 (46.9)	498 (47.2)	495 (46.6)	494 (46.7)	499 (47.1)	508 (47.7)	485 (46.1)	13,705 (43.6)	6813 (43.3)	6892 (43.8)	6878 (43.7)	6827 (43.4)
Other	382 (1.1)	35 (1.7)	15 (1.4)	20 (1.9)	15 (1.4)	20 (1.9)	19 (1.8)	16 (1.5)	347 (1.1)	173 (1.1)	174 (1.1)	185 (1.2)	162 (1.0)
Unknown	2238 (6.7)	63 (3.0)	31 (2.9)	32 (3.0)	28 (2.6)	35 (3.3)	34 (3.2)	29 (2.8)	2175 (6.9)	1103 (7.0)	1072 (6.8)	1089 (6.9)	1086 (6.9)
CHC^c^, n (%)													
CHC_1	22,430 (66.8)	1306 (61.7)	647 (61.3)	659 (62.1)	652 (61.7)	654 (61.7)	657 (61.6)	649 (61.8)	21,124 (67.2)	10,561 (67.2)	10,563 (67.2)	10,565 (67.2)	10,559 (67.2)
CHC_2	3809 (11.4)	278 (13.1)	141 (13.4)	137 (12.9)	137 (13.0)	141 (13.3)	140 (13.1)	138 (13.1)	3531 (11.2)	1768 (11.2)	1763 (11.2)	1765 (11.2)	1766 (11.2)
CHC_3	7317 (21.8)	533 (25.2)	267 (25.3)	266 (25.0)	268 (25.4)	265 (25.0)	269 (25.2)	264 (25.1)	6784 (21.6)	3392 (21.6)	3392 (21.6)	3392 (21.6)	3392 (21.6)
SCALE-UP I randomization, n (%)													
Not enrolled in SCALE-UP I	11,410 (34.0)	664 (31.4)	333 (31.6)	331 (31.2)	327 (30.9)	337 (31.8)	338 (31.7)	326 (31.0)	10,746 (34.2)	5377 (34.2)	5369 (34.2)	5376 (34.2)	5370 (34.2)
TM	9665 (28.8)	641 (30.3)	319 (30.2)	322 (30.3)	321 (30.4)	320 (30.2)	321 (30.1)	320 (30.4)	9024 (28.7)	4508 (28.7)	4516 (28.7)	4513 (28.7)	4511 (28.7)
TM+PN	12,481 (37.2)	812 (38.4)	403 (38.2)	409 (38.5)	409 (38.7)	403 (38.0)	407 (38.2)	405 (38.5)	11,669 (37.1)	5836 (37.1)	5833 (37.1)	5833 (37.1)	5836 (37.1)
Insurance status, n (%)													
Private	7916 (23.6)	610 (28.8)	299 (28.3)	311 (29.3)	331 (31.3)	279 (26.3)	310 (29.1)	300 (28.5)	7306 (23.2)	3650 (23.2)	3656 (23.3)	3630 (23.1)	3676 (23.4)
Public	5094 (15.2)	343 (16.2)	172 (16.3)	171 (16.1)	169 (16.0)	174 (16.4)	163 (15.3)	180 (17.1)	4751 (15.1)	2353 (15.0)	2398 (15.3)	2393 (15.2)	2358 (15.0)
Uninsured	20,546 (61.2)	1,164 (55.0)	584 (55.4)	580 (54.6)	557 (52.7)	607 (57.3)	593 (55.6)	571 (54.3)	19,382 (61.6)	9718 (61.8)	9664 (61.5)	9699 (61.7)	9683 (61.6)

a PN: patient navigation

b TM: text message

c CHC: community health center

### Primary Analyses—Request for at-Home COVID-19 Testing (Reach-Accept Testing)

A total of 2341/33,556 (7%) participants in both studies, 729/2117 (34.4%) in the smartphone study, and 1612/31,439 (5.1%) in the nonsmartphone study requested a test kit over the course of the trial.

#### Smartphone Study

Reach-Accept testing in the Chatbot arm was lower than in SMS text messaging (174/1051, 16.6% vs 555/1066, 52.1%; aRR 0.317, 98.33% CI 0.27‐0.38; *P*<.001; Table 1). No statistically significant differences in Reach-Accept testing were found between participants receiving messages every 10 days versus 30 days (381/1060, 35.9% vs 348/1057, 32.9%; aRR 1.088, 98.33% CI 0.96‐1.24; *P*=.12) or with access to PN versus no PN (360/1062, 33.9% vs 369/1055, 35.0%; aRR 0.976, 98.33% CI 0.86‐1.11; *P*=.65).

#### Nonsmartphone Study

Reach-Accept testing was higher among participants messaged every 10 days vs every 30 days (860/15,717, 5.5% vs 752/15,722, 4.8%; aRR 1.144, 97.5% CI 1.03‐1.28; *P*=.01; Table 2), and lower if the participants were offered access to PN compared with those in the no PN condition (680/15,718, 4.3% vs 932/15,721, 5.9%; aRR 0.729, 97.5% CI 0.65‐0.81; *P*<.001).

### Secondary and Implementation Outcomes

#### Smartphone Study

Out of 2117 participants in the smartphone study, 195 (9.2%) reported having used a test at least once (Multimedia Appendix 1, Table S1). Reach-Engage was lower for participants in chatbot versus SMS text messaging (309/1051, 29.4% vs 625/1066, 58.6%; aRR 0.502, 98.33% CI 0.44‐0.57; *P*<.001; Table 1). Participants were more likely to opt out from receiving further messages if they were in the 10-day versus 30-day condition (494/1060, 7.5% vs 440/1057, 2.6%; aRR 2.857, 98.33% CI 1.70‐4.80; *P*<.001). Of 1062 participants assigned to PN, 10 (0.9%) requested PN (PN-Request) and 6 (0.6%) answered the patient navigator’s call (PN-Engage; Multimedia Appendix 1, Table S2).

#### Nonsmartphone Study

Out of 33,556 participants in the Nonsmartphone study, 315/33,556 (0.9%) reported having used a test at least once (Multimedia Appendix 1). Reach-Engage was higher for participants in the 10-day versus 30-day condition (1388/15,717, 8.8% vs 1191/15,722, 7.6%; aRR 1.166, 97.5% CI 1.07‐1.27; *P*<.001; Table 2), and lower in the PN versus no PN condition (1157/15,718, 7.4% vs 1422/15,721, 9.0%; aRR 0.813, 97.5% CI 0.75‐0.89; *P*<.001). Of 15,718 participants assigned to PN, 19/15,718 (0.12%) requested PN (PN-Request) and 15/15,718 (0.10%) talked to a patient navigator (PN-Engage). Participants were more likely to opt out if they were in the 10-day versus 30-day arm (1977/15,717, 12.6% vs 1147/15,722, 7.3%; aRR 1.72, 97.5% CI 1.59‐1.86; *P*<.001). There were no statistically significant differences in the probability of opting out between participants randomized to PN vs no PN.

## Discussion

### Principal Findings

SCALE-UP II demonstrated the effectiveness of scalable, population-based intervention strategies in promoting the use of at-home COVID-19 testing among populations, including large proportions of patients who are Latino, low SES, and rural residents. Notably, 15,080/33,556 (44.9%) patients in the trial had a preference for a language other than English recorded in the clinic EHR, highlighting the critical need to provide interventions in English or Spanish, according to the patient’s preference. Overall, 37,597/43,325, 86.8% of eligible CHC patients had a valid, nonduplicate cellphone number in the EHR, and of those patients, 2341/33,556 (7%) requested a test kit at least once. This rate is higher than most monthly rates in a national survey of self-reported at-home testing across all demographic groups, which ranged from 2% in October 2021 to 7.5% in March 2022 [11]. Thus, the results suggest the effectiveness and potential use of SMS text messaging-based interventions for rapid public health response to pressing issues such as epidemics.

As hypothesized, patients randomized to receive messages every 10 days versus 30 days were more likely to request a kit (860/15,717, 5.5% vs 752/15,722, 4.8% in the nonsmartphone study). However, patients who received messages more frequently were also more likely to opt out from study interventions (494/1060, 7.5% vs 440/1057, 2.6% in the smartphone study and 977/15,717, 12.6% vs 1147/15,722, 7.3% in the nonsmartphone study), which is consistent with findings reported by previous studies using SMS text messaging-based interventions [30,31]. Thus, although the absolute proportion of patients who engaged and requested testing was greater in the higher frequency approach, the potential disadvantages of the larger opt-out rate would need to be considered based on context. Studies are needed to identify optimal dose-response versus opt-out trade-offs for various conditions, since findings may be different for topics such as health behaviors and preventive care. Future studies should also investigate approaches to minimize message volume, for example, by bundling communication about multiple preventive care measures (eg, breast and colorectal cancer screening and shingles vaccination) into a single message.

Unexpectedly, patients in the chatbot condition were less likely to request a test kit than those in the SMS text messaging condition (174/1051, 16.6% vs 555/1066, 52.1%), even though the chatbot offered several theoretical benefits such as more education about testing, a graphical user interface, and the ability to interact by clicking on buttons rather than typing. In addition, usability assessments conducted as a part of chatbot design showed that the chatbot was easy to use and acceptable. However, only 29.4% (309/1051) of the patients randomized to chatbot clicked on the hyperlink to launch the chatbot, while among those who launched the chatbot, 56.3% (174/309) requested a test. Therefore, it is possible that the extra steps in the chatbot arm before a test could be requested, including potential concerns (eg, fraud) with clicking on a hyperlink, imposed important engagement barriers. On the other hand, SMS text messaging offered an easy, 2-step option to request a test kit. Similar SMS text messaging-based approaches have shown higher efficacy in previous trials than more technically sophisticated tools such as patient portals [32,33]. It is also possible that the perceived value of the education that the chatbot provided was lower at the point of the pandemic when SCALE-UP II was launched, since the vast majority of patients had likely been exposed to extensive information about the COVID-19 pandemic or may have had strong opinions about pandemic response strategies, such as testing. These data suggest that the hyperlink step, rather than other chatbot-specific factors, introduced a significant barrier to engagement due to factors such as concerns about spam, limited data plan, and limited internet access. To address this issue, in ongoing and upcoming trials, we are delivering chatbot content via simple SMS text messaging rather than a web-based interface. This approach does not require internet access, does not require clicking on a hyperlink, and has a simpler user interface. Future studies will include more detailed analyses of predictors (eg, age, language preference, and rurality) of intervention engagement to help further elucidate this issue and inform digital health intervention design.

In the nonsmartphone study, patients in the PN condition were less likely to request a kit than those in no PN (680/15,718, 4.3% vs 932/15,721, 5.9%). This was also unexpected, since patients in the PN condition had to follow the exact same steps to request a kit as those in no PN but with the added option to talk to a patient navigator in case they had questions or needed help addressing barriers. In addition, there is substantial previous evidence demonstrating the effectiveness of PN in combination with digital health interventions improving the reach of screening and prevention behaviors [34-38]. It is not possible to conclude that talking to a patient navigator discouraged patients from requesting a test, since only 19/15,718 (0.12%) patients in the nonsmartphone study randomized to PN actually requested to speak with a navigator. These findings may be an idiosyncrasy of the COVID-19 pandemic, which became polarized with concerns about privacy and state surveillance. As such, patients in PN may have been concerned that a patient navigator would call them if they requested a test kit and ask questions about test results. Future studies should investigate more effective ways of providing PN without hindering engagement with a digital health tool, for example, offering access to a patient navigator only after patients have replied “YES” requesting a test kit. It is also possible that the chatbot and PN would have been more effective if the trial was conducted at earlier stages of the pandemic, when public health recommendations were more frequently changing, and people were less informed about COVID-19 testing.

### Implications for Research and Practice

SCALE-UP II interventions and findings are specific to the context of COVID-19 pandemic response. However, it is possible that some of the findings can be considered in the design of interventions for other population health interventions. For example, it is possible that for health-related behaviors that are widely known or familiar to patients, such as COVID testing was in the latter stages of the pandemic (eg, annual mammograms and flu shots), simple messaging interventions may be more effective than more complex educational approaches, such as a chatbot. Similarly, for urgent or time-sensitive behaviors, the trade-off of greater engagement versus greater drop-out may warrant frequent, repeated messaging. Finally, simple digital options for enacting positive health-related behaviors may actually outperform the options offering PN or guidance in some instances.

All of these possibilities require further investigation, and the reverse may be true for different behaviors and in different settings than what was found in the SCALE-UP II trial. For example, more complex and less familiar health-related behaviors, such as those that require shared decision-making, may benefit from greater education that can help patients make a more well-informed decision. Behaviors that are less time-sensitive could benefit from less frequent messaging that results in less drop-out, and the ability to speak with a community health worker or patient navigator may be superior for behaviors that are less familiar or require more complex actions. Further research is needed to tease out optimal strategies across the spectrum of health behaviors and contexts.

### Strengths and Limitations

Strengths of SCALE-UP II include a factorial design testing digital interventions with and without access to a human intervention, scalable and low-cost strategies to increase the uptake of COVID-19 testing through mailed test kits, and a large sample size with high reach to a diverse patient population that experiences health disparities. A particular strength is that this was a true population-level pragmatic trial where virtually all patients were automatically enrolled versus trials that require active patient consenting that can impose substantial selection biases and artificially influence engagement with study interventions.

The trial had important limitations. First, we were unable to reliably use the original primary outcome (ie, testing) due to a low response rate that was associated with treatment assignment and was likely associated with the outcome. For a reliable analysis, the missing at random assumption for multiple imputation would need to be very strong, stronger than we deemed realistic, to overcome the extent of missing outcomes. Nevertheless, requesting a COVID-19 test kit is an important outcome since patients may be more likely to test if they have a test kit readily available at home when needed. For example, a study assessing the use of the US COVIDTests.gov, which mailed free COVID-19 test kits to US citizens upon request through the program website, reported that 59.9% (77,089,010) of US households ordered kits; in 32.1% of households, at least 1 government test kit was used; and 23.6% were unlikely to have tested without COVIDTests.gov [34,39]. In addition, unlike other types of access to testing (eg, pharmacies and laboratories), testing rates through COVIDTests.gov were more similar across demographic groups. Therefore, individuals requesting mailed COVID-19 test kits are an important outcome that can produce equitable public health impact. Also, requesting a COVID-19 test kit is an important outcome since patients may be more likely to test if they have a test kit readily available at home when needed. A second limitation was that SCALE-UP II was launched after most of the US population had been vaccinated or had been infected by COVID-19, so it is possible that patients’ motivation to get tested and receive information about COVID-19 was lower than in earlier stages of the pandemic.

### Conclusions

SCALE-UP II tested the effect of digital health interventions on the uptake of distribution of at-home COVID-19 test kits among patients from populations receiving care at CHC settings. A simple intervention using bidirectional SMS text messaging was more effective than a more complex chatbot on increasing the reach of COVID-19 testing. Although messaging every 10 days was more effective than every 30 days, it also led to a larger opt-out rate, so the trade-offs between effectiveness and opt-out rate should be considered based on context. Digital health interventions based on automated bidirectional SMS text messaging in the patient’s preferred language are a simple, scalable, and low-cost strategy to offer access to at-home COVID-19 testing to those populations. Similar approaches may be used to support public health response and other forms of at-home testing.

## Supplementary material

10.2196/74145 Multimedia Appendix 1 Online supplement with additional figures and tables.

10.2196/74145 Checklist 1 Consolidated Standards of Reporting Trials checklist.

## Abbreviations

aRR: adjusted risk ratios
AUCH: Association for Utah Community Health
CHC: community health center
CONSORT: Consolidated Standards of Reporting Trials
CPSTF: Community Preventive Services Task Force
EHR: electronic health record
PN: patient navigation
SES: low socioeconomic status
