# Supplementary material for: Population-Based Digital Health Interventions to Deliver at-Home COVID-19 Testing: SCALE-UP II Randomized Clinical Trial
Source: J Med Internet Res. 2025 Jul 28;27:e74145. doi: 10.2196/74145 (PMC12303405; doi:10.2196/74145)
Supplement: Multimedia Appendix 1 [file jmir-v27-e74145-s001.docx]

**Supplementary Materials**

Contents

[Interventions: Sample Screen Shots 2](#_Toc198309376)

[Introductory Message Script 3](#_Toc198309377)

[Smartphone study 3](#_Toc198309378)

[Non-Smartphone study 3](#_Toc198309379)

[Chatbot transcript (introduction and option to request a test) 4](#_Toc198309380)

[Self-reported testing rates 5](#_Toc198309381)

[PN-Request and PN-Engage 6](#_Toc198309382)

[Multivariate analysis results 7](#_Toc198309383)

[Preliminary analysis for the primary outcome: interactions included 7](#_Toc198309384)

[Preferred model: log-binomial model with no interactions 10](#_Toc198309385)

[Follow-up Response by Study Condition 14](#_Toc198309386)

# Interventions: Sample Screen Shots


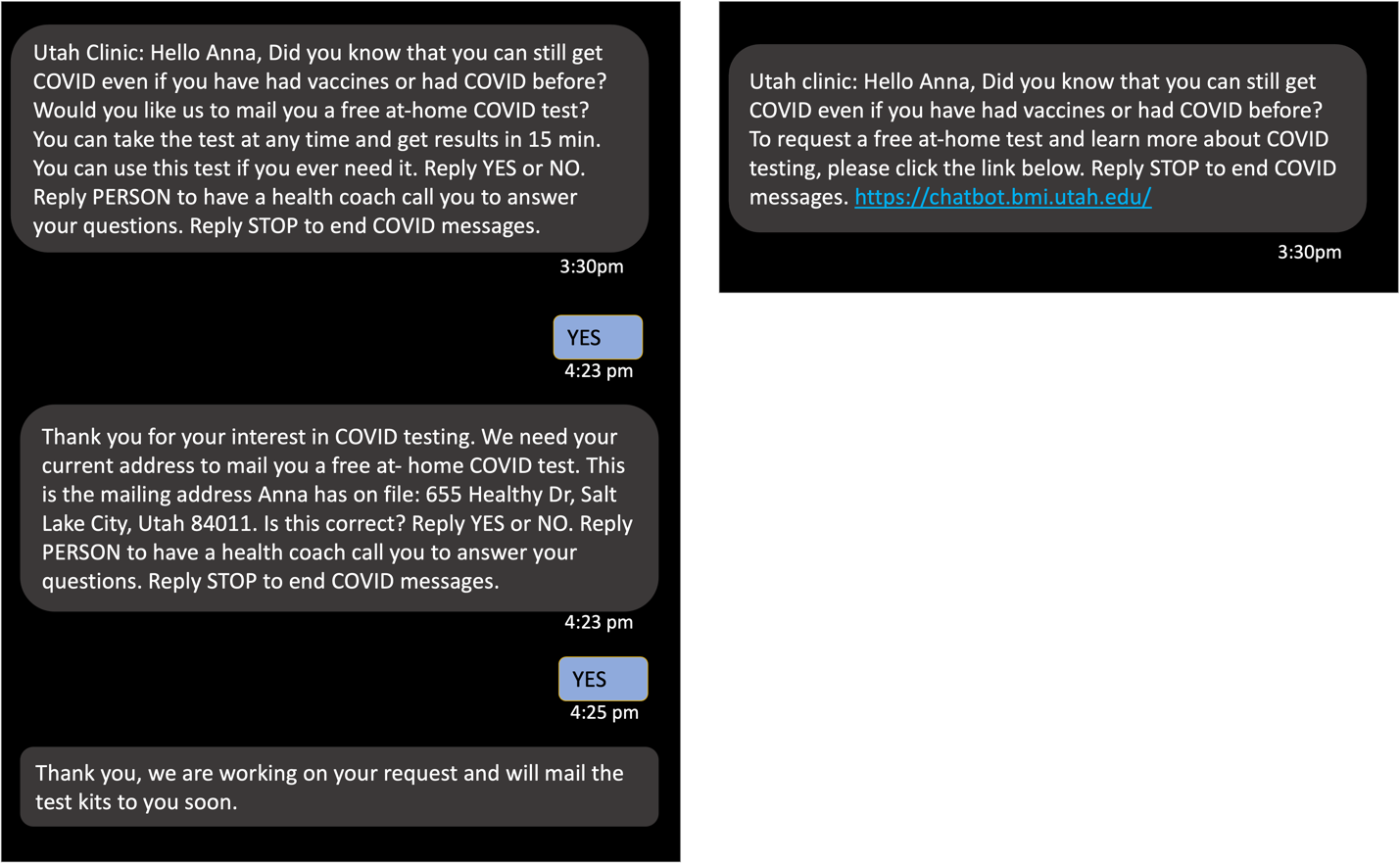


eFigure 1 – Text message sent to patients randomized to TM+PN with options to reply YES to request at-home COVID-19 test kits or PERSON to request to receive a call from a patient navigator (left); and text message sent to patients randomized to Chatbot with a hyperlink to launch the chatbot (right).


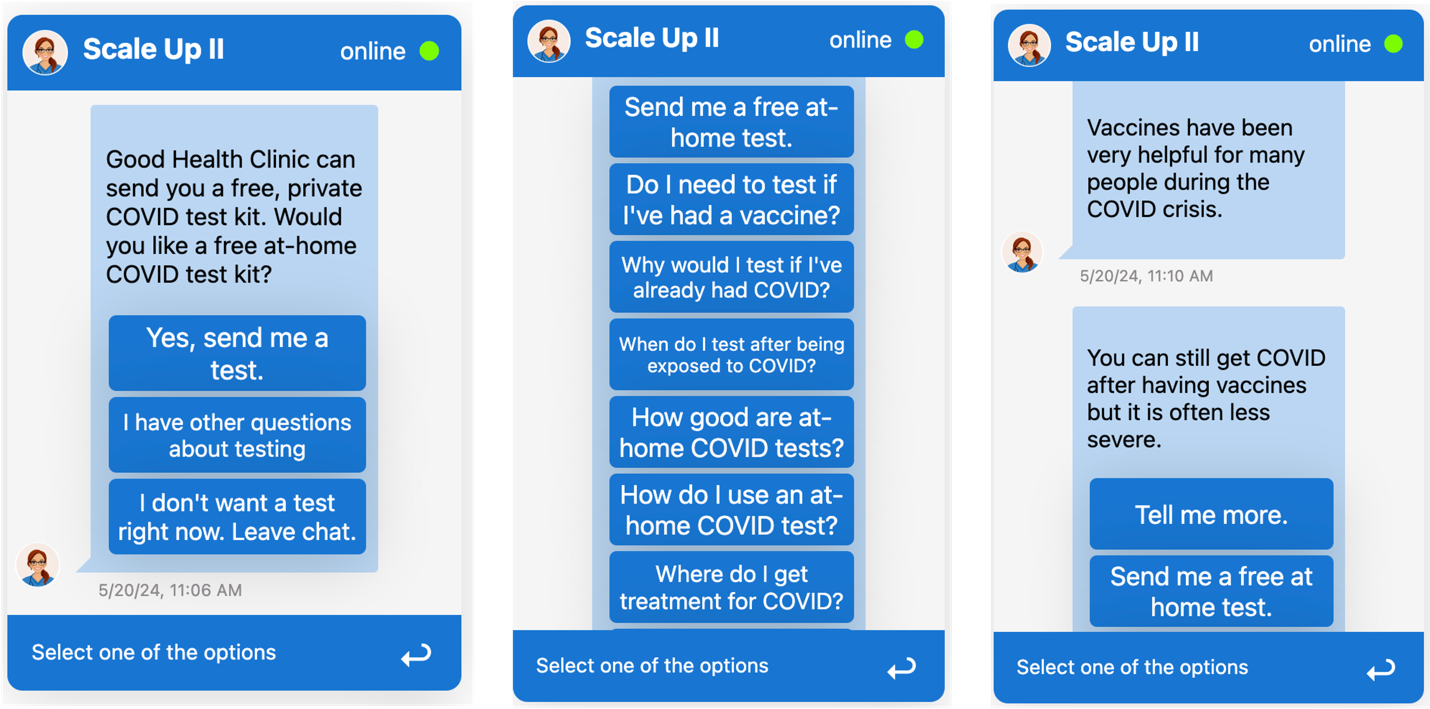


eFigure 2 – Screenshots of Chatbot interaction with the initial introduction (left), the list of questions that patients could ask (middle), and information related to the question about testing for those who have been vaccinated (right).

# Introductory Message Script

Script of the intervention messages texted to the participants by study arm and by language in the Smartphone study and Non-Smartphone study.

## Smartphone study

| Arm | English | Spanish |
| --- | --- | --- |
| CB and CB+PN | "[*CHC name*]: Hello [*patient’s first name*], Did you know that you can still get COVID even if you have had vaccines or had COVID before? To request a free at-home test and learn more about COVID testing, please click the link below. Reply STOP to end COVID messages." | "[CHC name]: Hola [*patient’s first name*], ¿Sabía que aún puede contraer COVID incluso si ha recibido vacunas o ha tenido COVID antes? Para solicitar una prueba gratuita en el hogar y obtener más información sobre las pruebas de COVID, haga clic en el siguiente enlace. Responda ALTO para parar los mensajes de COVID." |

## Non-Smartphone study

| Arm | English | Spanish |
| --- | --- | --- |
| TM+PN | "$[*CHC name*]: Hello [*patient’s first name*], Did you know that you can still get COVID even if you have had vaccines or had COVID before? Would you like us to mail you a free at-home COVID test? A self-test you can take at any time and get results in 15 minutes. You can use this test if you ever need it. Reply YES or NO. Reply PERSON to have a health coach call you to answer your questions. Reply STOP to end COVID messages." | "[*CHC name*]: Hola [*patient’s first name*]: ¿Sabía que aún puede contraer COVID incluso si se ha vacunado o ha tenido COVID antes? ¿Le gustaría que le enviemos por correo una prueba gratuita de COVID en casa? Una prueba que puede realizar en cualquier momento y obtener resultados en 15 minutos. Puede usar esta prueba si cuando lo necesite. Responda SI o NO. Responda PERSONA para que una guía de salud lo llame y responda sus preguntas. Responda ALTO para finalizar los mensajes de COVID." |
| TM only | "$[*CHC name*]: Hello [*patient’s first name*], Did you know that you can still get COVID even if you have had vaccines or had COVID before? Would you like us to mail you a free at-home COVID test? A self-test you can take at any time and get results in 15 minutes. You can use this test if you ever need it. Reply YES or NO. Reply STOP to end COVID messages." | "$[*CHC name*]: Hola [*patient’s first name*]: ¿Sabía que aún puede contraer COVID incluso si se ha vacunado o ha tenido COVID antes? ¿Le gustaría que le enviemos por correo una prueba gratuita de COVID en casa? Una prueba que puede realizar en cualquier momento y obtener resultados en 15 minutos. Puede usar esta prueba si cuando lo necesite. Responda SI o NO. Responda ALTO para finalizar los mensajes de COVID." |

* Differences are highlighted in yellow.

# Chatbot transcript (introduction and option to request a test)

Chatbot: Hi there! I'm Sam, a virtual assistant, from [*CHC name*], here to help you order a free at-home COVID test kit. I can also answer your questions about COVID testing.

Chatbot: It's easy for us to chat if you click on one of the choices shown after I send you a message. Try it here by pressing this response:

Got it!

Chatbot: Great job! You've got it.

Chatbot: You may want to keep a free COVID test kit at home in case you need it. If you have COVID, it is important to find out early so you can get treatment from the clinic.

Chatbot: [*CHC name*] can send you a free, private COVID test kit. Would you like an at-home COVID test kit?

Yes, send me a test.

!

Person, have someone call me.

!

I have other questions about testing

!

I don't want a test right now. Leave chat.

!

# Self-reported testing rates

Table S1. Follow-up response and self-reported testing rates (only for participants who requested COVID-19 test kits).

|  | **Both studies** | **Smartphone study** | | | | | | | **Non-Smartphone study** | | | | |
| --- | --- | --- | --- | --- | --- | --- | --- | --- | --- | --- | --- | --- | --- |
|  |  | **Overall** | **Patient navigation** | | **Outreach frequency** | | **Delivery mechanism** | | **Overall** | **Patient navigation** | | **Outreach frequency** | |
|  |  |  | No PN | PN | 30 days | 10 days | TM | Chatbot |  | No PN | PN | 30 days | 10 days |
| Total: N of patients who ordered tests | 2,341 | 729 | 369 | 360 | 348 | 381 | 555 | 174 | 1,612 | 932 | 680 | 752 | 860 |
| Responded to follow-up (response rate) | 1,459 (62.3%) | 532 (73.0%) | 278 (75.3%) | 254 (70.6%) | 250 (71.8%) | 282 (74.0%) | 395 (71.2%) | 137 (78.7%) | 927 (57.5%) | 531 (57.0%) | 396 (58.2%) | 447 (59.4%) | 480 (55.8%) |
| Used a COVID-19 at-home test (testing rate) | 510 (35.0%) | 195 (36.7%) | 103 (37.1%) | 92 (36.2%) | 91 (36.4%) | 104 (36.9%) | 135 (34.2%) | 60 (43.8%) | 315 (34.0%) | 180 (33.9%) | 135 (34.1%) | 158 (35.3%) | 157 (32.7%) |

# PN-Request and PN-Engage

Table S2. PN-Request and PN-Engage (only for participants who were randomized to the PN condition).

|  | **Smartphone study** | **Non-Smartphone study** |
| --- | --- | --- |
| Total N in the PN condition | 1,062 | 15,718 |
| PN-Request, n (%) | 10 (0.9%) | 19 (0.1%) |
| PN-Engage, n (%) | 6 (0.6%) | 15 (0.1%) |

# Multivariate analysis results

### Preliminary analysis for the primary outcome: interactions included

As specified in the Scale-Up II protocol paper, we preliminarily estimated a model specification including the pairwise and 3-way interactions between the main effects and the smartphone ownership indicator in order to assess for any synergistic and/or antagonistic effect modifications across interventions. We have conducted these analyses for the primary outcome (Reach-Accept Testing) for both the smartphone and non-smartphone groups. In both analyses, the estimated coefficients on all interaction terms were not statistically significant at the 5% level. We have conducted an omnibus test (using the car::linearHypothesis function in R) and found that in each case, these coefficients were jointly not statistically significant.

Table S3. Estimation results: log binomial model with interaction terms (dependent variable is *Reach-Accept Testing*).

|  | Smartphone study, N = 2,117 | | | Non-Smartphone study, N = 31,439 | | |
| --- | --- | --- | --- | --- | --- | --- |
| Explanatory variables | Risk ratio | 95% CI for RR | *P-value* | Risk ratio | 95% CI for RR | *P-value* |
| PN arm | 0.899 | (0.758, 1.067) | .22 | 0.778 | (0.676, 0.896) | <.001 |
| 10-day arm | 1.022 | (0.871, 1.200) | .78 | 1.204 | (1.062, 1.364) | .003 |
| Chatbot arm | 0.309 | (0.229, 0.417) | <.0001 |  |  |  |
| PN*10day | 1.169 | (0.929, 1.471) | .18 | 0.886 | (0.730, 1.074) | .21 |
| PN*Chatbot | 1.168 | (0.767, 1.778) | .46 |  |  |  |
| 10day*Chatbot | 1.073 | (0.711, 1.619) | .73 |  |  |  |
| PN*10day*Chatbot | 0.714 | (0.396, 1.288) | .26 |  |  |  |
| Rural | 1.100 | (0.766, 1.580) | .60 | 1.690 | (1.281, 2.229) | <.001 |
| SCALE-UP I randomization (reference category: not enrolled in SCALE-UP I) | | | | | | |
| TM | 1.114 | (0.963, 1.290) | .14 | 1.285 | (1.126, 1.466) | <.001 |
| TMPN | 1.098 | (0.952, 1.265) | .19 | 1.159 | (1.019, 1.318) | .024 |
| CHC (reference category: CHC 1) | | | | | | |
| CHC 2 | 1.057 | (0.728, 1.535) | .77 | 0.786 | (0.589, 1.050) | .10 |
| CHC 3 | 0.940 | (0.816, 1.083) | .39 | 1.231 | (1.089, 1.392) | <.001 |
| Intervention start month (reference category: June 2023) | | | | | | |
| Dec 2022 | 0.636 | (0.197, 2.053) | .44 | 1.078 | (0.627, 1.855) | 0.78 |
| Feb 2023 | 1.047 | (0.724, 1.514) | .80 | 1.274 | (0.965, 1.681) | 0.08 |
| Mar 2024 | 1.158 | (0.988, 1.359) | .070 | 1.119 | (0.968, 1.293) | .129 |
| Apr 2023 | 1.109 | (0.960, 1.281) | .160 | 1.135 | (1.001, 1.286) | .047 |
| May 2023 | 1.119 | (0.958, 1.307) | .156 | 1.036 | (0.905, 1.187) | .606 |
| Intercept | 0.445 | (0.370, 0.535) | <0.001 | 0.041 | (0.035, 0.047) | <.0001 |

**Table S4 - Test for joint significance of the interaction terms.**

|  | **Patient group:** | |
| --- | --- | --- |
|  | **Smartphone study** | **Non-Smartphone study** |
| Number of patients | 2117 | 31,439 |
| Hypotheses tested | PN*chatbot = 0  PN*d10 = 0  Chatbot*d10 = 0  PN*chatbot*d10 = 0 | PN*d10 |
| Chi-sq statistics | 2.5984 | 1.5149 |
| Degrees of freedom | 4 | 1 |
| *P*-value | .62 | .21 |

In addition to the log-binomial model, we also estimated the same specification using logistics regression for a robustness check. The logistics regression results were similar to the ones obtained using the log-binomial model (see Table S5).

Table S5. Estimation results: logistic regression model with interaction terms (dependent variable is *Reach-Accept Testing*).

|  | **Smartphone study, N = 2,117** | | | **Non-Smartphone study, N = 31,439** | | |
| --- | --- | --- | --- | --- | --- | --- |
| **Explanatory variables** | OR | 95% CI for OR | *P*-value | OR | 95% CI for OR | *P*-value |
| PN arm | 0.770 | (0.546, 1.085) | .13 | 0.768 | (0.662, 0.889) | <0.001 |
| 10-day arm | 1.039 | (0.737, 1.465) | .82 | 1.218 | (1.067, 1.392) | .003 |
| Chatbot arm | 0.164 | (0.107, 0.245) | <.001 |  |  |  |
| PN*10day | 1.439 | (0.887, 2.339) | .14 | 0.879 | (0.717, 1.077) | .21 |
| PN*Chatbot | 1.461 | (0.821, 2.604) | .19 |  |  |  |
| 10day*Chatbot | 1.142 | (0.643, 2.030) | .65 |  |  |  |
| PN*10day*Chatbot | 0.522 | (0.231, 1.176) | .11 |  |  |  |
| Rural | 1.238 | (0.618, 2.501) | .54 | 1.768 | (1.315, 2.376) | <.001 |
| SCALE-UP I randomization (reference category: not enrolled in SCALE-UP I) | | | | | | |
| TM | 1.265 | (0.968, 1.656) | .08 | 1.301 | (1.132, 1.495) | <.001 |
| TMPN | 1.233 | (0.954, 1.596) | .11 | 1.166 | (1.019, 1.335) | .026 |
| CHC (reference category: CHC 1) | | | | | | |
| CHC 2 | 1.096 | (0.533, 2.226) | .80 | 0.763 | (0.560, 1.033) | .08 |
| CHC 3 | 0.941 | (0.730, 1.211) | .63 | 1.248 | (1.095, 1.420) | <.001 |
| Intervention start month (reference category: June 2023) | | | | | | |
| Dec 2022 | 0.449 | (0.063, 2.158) | .34 | 1.099 | (0.587, 1.908) | 0.75 |
| startInt_2023-02 | 1.084 | (0.569, 2.019) | .80 | 1.296 | (0.954, 1.734) | 0.08 |
| startInt_2023-03 | 1.377 | (1.025, 1.849) | .03 | 1.126 | (0.965, 1.310) | 0.12 |
| startInt_2023-04 | 1.222 | (0.942, 1.586) | .13 | 1.143 | (1.002, 1.304) | 0.04 |
| startInt_2023-05 | 1.233 | (0.929, 1.636) | .14 | 1.038 | (0.899, 1.197) | 0.60 |
| (Intercept) | 0.792 | (0.559, 1.121) | .18 | 0.043 | (0.036, 0.050) | <.001 |

### Preferred model: log-binomial model with no interactions

Since the preliminary analyses did not support the inclusion of the interaction terms, they were excluded from the preferred model specification. The tables below contain the full results for the log-binomial model analysis for the primary and secondary outcomes, including the coefficient estimates on covariates (rural vs. urban, CHC, randomization arm in the previous SCALE-UP I trial, and a categorical variable reflecting the calendar month the participant received the first intervention message).

Table S6. Primary and secondary outcomes for the Smartphone study.

|  | Total N in group | **Reach-Accept Testing** | | | | | **Reach-Engage** | | | | | **Opt-out** | | | | |
| --- | --- | --- | --- | --- | --- | --- | --- | --- | --- | --- | --- | --- | --- | --- | --- | --- |
|  |  | n | % | RR | 98.3% CI | *P-*value | n | % | RR | 98.33% CI | *P*-value | n | % | RR | 98.3% CI | *P*-value |
|  | (1) | (2) | (3) | (4) | (5) | (6) | (7) | (8) | (9) | (10) | (11) | (12) | (13) | (14) | (15) | (16) |
| **Patient Navigation (PN)** |  |  |  |  |  |  |  |  |  |  |  |  |  |  |  |  |
| PN not offered [Ref] | 1,055 | 369 | 35.0% |  |  |  | 472 | 44.7% |  |  |  | 42 | 4.0% |  |  |  |
| PN offered | 1,062 | 360 | 33.9% | 0.976 | (0.86, 1.11) | .65 | 462 | 43.5% | 0.998 | (0.90, 1.11) | .96 | 64 | 6.0% | 1.463 | (0.93, 2.30) | .045 |
| **Outreach frequency** |  |  |  |  |  |  |  |  |  |  |  |  |  |  |  |  |
| Every 30 days [Ref] | 1,057 | 348 | 32.9% |  |  |  | 440 | 41.6% |  |  |  | 27 | 2.6% |  |  |  |
| Every 10 days | 1,060 | 381 | 35.9% | 1.088 | (0.96, 1.24) | .12 | 494 | 46.6% | 1.099 | (0.99, 1.22) | .036 | 79 | 7.5% | 2.857 | (1.70, 4.80) | <0.001 |
| **Delivery mechanism** |  |  |  |  |  |  |  |  |  |  |  |  |  |  |  |  |
| TM [Ref] | 1,066 | 555 | 52.1% |  |  |  | 625 | 58.6% |  |  |  | 50 | 4.7% |  |  |  |
| Chatbot | 1,051 | 174 | 16.6% | 0.317 | (0.27, 0.38) | p<.001 | 309 | 29.4% | 0.502 | (0.44, 0.57) | <.001 | 56 | 5.3% | 1.172 | (0.75, 1.83) | .39 |
| **CHC** |  |  |  |  |  |  |  |  |  |  |  |  |  |  |  |  |
| CHC_1 [Ref] | 1,306 | 448 | 34.3% |  |  |  | 575 | 44.0% |  |  |  | 55 | 4.2% |  |  |  |
| CHC_2 | 278 | 105 | 37.8% | 1.062 | (0.67, 1.67) | .75 | 136 | 48.9% | 1.211 | (0.83, 1.76) | .22 | 31 | 11.2% | 1.690 | (0.45, 6.38) | .34 |
| CHC_3 | 533 | 176 | 33.0% | 0.940 | (0.79, 1.12) | .39 | 223 | 41.8% | 0.920 | (0.79, 1.07) | .17 | 20 | 3.8% | 0.955 | (0.49, 1.85) | .87 |
| **Intervention start** |  |  |  |  |  |  |  |  |  |  |  |  |  |  |  |  |
| Dec 2022 | 7 | 2 | 28.6% | 0.650 | (0.15, 2.74) | .47 | 2 | 28.6% | 0.595 | (0.14, 2.47) | .38 | 1 | 14.3% | 2.613 | (0.27, 25.52) | .31 |
| Feb 2023 | 64 | 20 | 31.3% | 1.049 | (0.67, 1.65) | .80 | 26 | 40.6% | 1.067 | (0.73, 1.56) | .68 | 2 | 3.1% | 0.585 | (0.10, 3.51) | .47 |
| Mar 2023 | 391 | 146 | 37.3% | 1.162 | (0.96, 1.41) | .06 | 184 | 47.1% | 1.117 | (0.95, 1.32) | .11 | 13 | 3.3% | 0.497 | (0.24, 1.05) | .025 |
| Apr 2023 | 622 | 220 | 35.4% | 1.113 | (0.93, 1.33) | .15 | 283 | 45.5% | 1.103 | (0.95, 1.28) | .11 | 29 | 4.7% | 0.659 | (0.38, 1.15) | .074 |
| May 2023 | 454 | 159 | 35.0% | 1.118 | (0.92, 1.35) | .16 | 204 | 44.9% | 1.110 | (0.95, 1.30) | .11 | 22 | 4.8% | 0.720 | (0.39, 1.32) | .20 |
| June 2023 [Ref] | 579 | 182 | 31.4% |  |  |  | 235 | 40.6% |  |  |  | 39 | 6.7% |  |  |  |
| **SU-1 randomization** |  |  |  |  |  |  |  |  |  |  |  |  |  |  |  |  |
| Not in SU-1 [Ref] | 664 | 219 | 33.0% |  |  |  | 272 | 41.0% |  |  |  | 53 | 8.0% |  |  |  |
| TM | 641 | 228 | 35.6% | 1.114 | (0.93, 1.33) | .15 | 294 | 45.9% | 1.175 | (1.01, 1.37) | .010 | 26 | 4.1% | 0.649 | (0.35, 1.19) | .087 |
| TMPN | 812 | 282 | 34.7% | 1.094 | (0.92, 1.30) | .22 | 368 | 45.3% | 1.176 | (1.02, 1.36) | .008 | 27 | 3.3% | 0.555 | (0.30, 1.02) | .02 |
| **Urban vs Rural** |  |  |  |  |  |  |  |  |  |  |  |  |  |  |  |  |
| Rural | 277 | 107 | 38.6% | 1.100 | (0.71, 1.71) | .61 | 135 | 48.7% | 0.998 | (0.69, 1.44) | .99 | 30 | 10.8% | 1.222 | (0.33, 4.53) | .71 |
| Urban [Ref] | 1,840 | 622 | 33.8% |  |  |  | 799 | 43.4% |  |  |  | 76 | 4.1% |  |  |  |
| **Intercept** |  |  |  | 0.431 | (0.35, 0.53) | <.001 |  |  | 0.461 | (0.38, 0.55) | <.001 |  |  | 0.031 | (0.01, 0.07) | <.001 |

Columns (2) and (3) report the number of participants who requested test kits (Reach-Accept Testing) and their share among those randomized in that arm, respectively. Columns (7) and (8) report similar descriptive statistics for Reach-Engage, and columns (12) and (13) – for Opt-out.

For the Smartphone study, alpha was set to 0.0167 and 98.33% confidence intervals were calculated to account for three co-primary comparisons. For the Non-Smartphone study, alpha was set to 0.025 and 97.5% confidence intervals were calculated to account for two co-primary comparisons.

Table S7. Primary and secondary outcomes for the Non-Smartphone study.

|  | Total N in group | **Reach-Accept Testing** | | | | | **Reach-Engage** | | | | | **Opt-out** | | | | |
| --- | --- | --- | --- | --- | --- | --- | --- | --- | --- | --- | --- | --- | --- | --- | --- | --- |
|  |  | n | % | RR | 97.5% CI | *P*-value | n | % | RR | 97.5% CI | *P*-value | n | % | RR | 97.5% CI | *P*-value |
|  | (1) | (2) | (3) | (4) | (5) | (6) | (7) | (8) | (9) | (10) | (11) | (12) | (13) | (14) | (15) | (16) |
| **Patient Navigation (PN)** |  |  |  |  |  |  |  |  |  |  |  |  |  |  |  |  |
| PN not offered [Ref] | 15,721 | 932 | 5.9% |  |  |  | 1,422 | 9.0% |  |  |  | 1,565 | 10.0% |  |  |  |
| PN offered | 15,718 | 680 | 4.3% | 0.729 | (0.65, 0.81) | <.001 | 1,157 | 7.4% | 0.813 | (0.75, 0.89) | <.001 | 1,559 | 9.9% | 0.996 | (0.92, 1.07) | .90 |
| **Outreach frequency** |  |  |  |  |  |  |  |  |  |  |  |  |  |  |  |  |
| Every 30 days [Ref] | 15,722 | 752 | 4.8% |  |  |  | 1,191 | 7.6% |  |  |  | 1,147 | 7.3% |  |  |  |
| Every 10 days | 15,717 | 860 | 5.5% | 1.144 | (1.03, 1.28) | .005 | 1,388 | 8.8% | 1.166 | (1.07, 1.27) | <.001 | 1,977 | 12.6% | 1.718 | (1.59, 1.86) | <.001 |
| **CHC** |  |  |  |  |  |  |  |  |  |  |  |  |  |  |  |  |
| CHC_1 [Ref] | 21,124 | 1,018 | 4.8% |  |  |  | 1,609 | 7.6% |  |  |  | 1,917 | 9.1% |  |  |  |
| CHC_2 | 3,531 | 179 | 5.1% | 0.787 | (0.57, 1.09) | .10 | 344 | 9.7% | 0.967 | (0.76, 1.23) | .76 | 763 | 21.6% | 2.028 | (1.73, 2.37) | <.001 |
| CHC_3 | 6,784 | 415 | 6.1% | 1.231 | (1.07, 1.42) | <.001 | 626 | 9.2% | 1.181 | (1.06, 1.32) | <.001 | 444 | 6.5% | 0.756 | (0.67, 0.85) | <.001 |
| **Intervention start** |  |  |  |  |  |  |  |  |  |  |  |  |  |  |  |  |
| Dec 2022 | 181 | 14 | 7.7% | 1.078 | (0.58, 2.00) | .79 | 20 | 11.0% | 1.013 | (0.61, 1.68) | .96 | 13 | 7.2% | 0.994 | (0.54, 1.84) | .98 |
| Feb 2023 | 838 | 59 | 7.0% | 1.274 | (0.93, 1.75) | .087 | 87 | 10.4% | 1.202 | (0.93, 1.55) | .11 | 51 | 6.1% | 0.879 | (0.63, 1.22) | .38 |
| Mar 2023 | 5,043 | 274 | 5.4% | 1.118 | (0.95, 1.32) | .13 | 431 | 8.5% | 1.076 | (0.95, 1.22) | .20 | 435 | 8.6% | 0.876 | (0.78, 0.99) | .013 |
| Apr 2023 | 8,139 | 437 | 5.4% | 1.135 | (0.98, 1.31) | .048 | 683 | 8.4% | 1.073 | (0.96, 1.20) | .15 | 808 | 9.9% | 0.940 | (0.85, 1.04) | .15 |
| May 2023 | 6,625 | 328 | 5.0% | 1.037 | (0.89, 1.21) | .61 | 533 | 8.0% | 1.017 | (0.90, 1.15) | .75 | 709 | 10.7% | 0.989 | (0.89, 1.09) | .81 |
| June 2023 [Ref] | 10,613 | 500 | 4.7% |  |  |  | 825 | 7.8% |  |  |  | 1,108 | 10.4% |  |  |  |
| **SU-1 randomization** |  |  |  |  |  |  |  |  |  |  |  |  |  |  |  |  |
| Not in SU-1 [Ref] | 10,746 | 491 | 4.6% |  |  |  | 851 | 7.9% |  |  |  | 1,483 | 13.8% |  |  |  |
| TM | 9,024 | 505 | 5.6% | 1.284 | (1.10, 1.49) | <.001 | 767 | 8.5% | 1.188 | (1.06, 1.34) | .001 | 753 | 8.3% | 0.789 | (0.71, 0.88) | <.001 |
| TMPN | 11,669 | 616 | 5.3% | 1.159 | (1.00, 1.34) | .02 | 961 | 8.2% | 1.113 | (0.99, 1.25) | .035 | 888 | 7.6% | 0.760 | (0.69, 0.84) | <.001 |
| **Urban vs Rural** |  |  |  |  |  |  |  |  |  |  |  |  |  |  |  |  |
| Rural | 3,063 | 182 | 5.9% | 1.690 | (1.23, 2.32) | <.001 | 334 | 10.9% | 1.598 | (1.26, 2.02) | <.001 | 617 | 20.1% | 1.000 | (0.85, 1.17) | .99 |
| Urban [Ref] | 28,376 | 1,430 | 5.0% |  |  |  | 2,245 | 7.9% |  |  |  | 2,507 | 8.8% |  |  |  |
| **Intercept** |  |  |  | 0.042 | (0.04, 0.05) | <.001 |  |  | 0.067 | (0.06, 0.08) | <.001 |  |  | 0.083 | (0.07, 0.09) | <.001 |

Columns (2) and (3) report the number of participants who requested test kits (Reach-Accept Testing) and their share among those randomized in that arm, respectively. Columns (7) and (8) report similar descriptive statistics for Reach-Engage, and columns (12) and (13) – for Opt-out.

For the Smartphone study, alpha was set to 0.0167 and 98.33% confidence intervals were calculated to account for three co-primary comparisons. For the Non-Smartphone study, alpha was set to 0.025 and 97.5% confidence intervals were calculated to account for two co-primary comparisons.

# Follow-up Response by Study Condition

This section examines the effect of the study conditions, i.e. delivery mechanism (TM vs. chatbot), outreach frequency (every 10 vs. 30 days), and PN (with and without access to PN) on *Follow-Up Response*, i.e. the proportion of study participants who responded to at least one of the follow-up messages after the end of the intervention and reported their *Testing* outcome. These proportions were calculated both among all randomized participants and only among those who received follow-up messages and/or surveys asking to report testing outcomes. Participants who opted out at any point during the intervention were not contacted at the follow-up stage. In addition, a small number of participants who did not opt out were not sent follow-up messages due to a software error; in the Smartphone study, 67 patients (3.1% of the total number of participants) were affected by this issue, and in the Non-Smartphone Study, 56 patients (0.2%) were affected.

Log-binomial models were used to regress *Follow-Up Response* upon each of the three main effects in the Smartphone study, i.e. Chatbot (vs. TM), PN (vs. No PN), and outreach frequency (10 vs. 30 days); and two main effects in the Non-Smartphone study, i.e. PN (vs. No PN) and outreach frequency (10 vs. 30 days). The estimates from these models, along with the follow-up response rates by study condition, are reported in Table S8 (Smartphone Study) and Table S9 (Non-Smartphone Study).

In the Smartphone Study, *Follow-Up Response among all randomized participants* in the Chatbot arm was *lower* than in TM (34.7% vs. 46.6%, aRR=0.745 [95% CI 0.67-0.83], P<.0001). In the Non-Smartphone Study, *Follow-Up Response among all randomized participants* was *lower* among participants messaged every 10 days vs. every 30 days (6.0% vs 7.4%, aRR=0.811 [95% CI 0.75-0.88], P<.0001), and *lower* if the participants were offered access to PN compared to those in the No PN condition (6.2% vs 7.2%, aRR=0. 862 [95% CI 0.79, 0.94], P=.0004). The results were nearly identical when the response rate was calculated as a proportion of study participants who responded to follow-up messages among those who received follow-up messages (Panel B in Table S8 and Table S9). Overall, these findings suggest that participants randomized to conditions with lower *Reach-Accept Test* (primary outcome), or higher opt-out rates had systematically lower follow-up response rates.

**Table S8. Follow-Up Response by Study Condition – Smartphone Study**

|  | ***Reported Testing* outcome:** | | | | | | | | | | | |
| --- | --- | --- | --- | --- | --- | --- | --- | --- | --- | --- | --- | --- |
|  | 1. **Among all randomized participants** | | | | | | 1. **Among participants who received follow-up messages** | | | | | |
|  | N | n | % | aRR | 95% CI | *P-value* | N | n | % | aRR | 95% CI | *P-value* |
| **Delivery mechanism** |  | | | | | |  |  | | | | |
| TM [Ref] | 1,066 | 497 | 46.6% |  |  |  | 1,014 | 497 | 49.0% |  |  |  |
| Chatbot | 1,051 | 365 | 34.7% | 0.745 | (0.67, 0.83) | <.0001 | 930 | 365 | 39.2% | 0.801 | (0.72, 0.89) | <0.0001 |
| **Outreach frequency** |  | | | | | |  |  | | | | |
| Every 30 days [Ref] | 1,057 | 449 | 42.5% |  |  |  | 979 | 449 | 45.9% |  |  |  |
| Every 10 days | 1,060 | 413 | 39.0% | 0.924 | (0.83, 1.02) | 0.13 | 965 | 413 | 42.8% | 0.947 | (0.86, 1.05) | 0.28 |
| **PN** |  | | | | | |  |  | | | | |
| PN not offered [Ref] | 1,055 | 443 | 42.0% |  |  |  | 977 | 443 | 45.3% |  |  |  |
| PN offered | 1,062 | 419 | 39.5% | 0.93 | (0.84, 1.03) | 0.16 | 967 | 419 | 43.3% | 0.944 | (0.85, 1.04) | 0.25 |

N = total number of participants in the group, n = number of patients who reported their testing outcome, aRR=adjusted risk ratio from the log-binomial model; CI=confidence interval; PN=Patient Navigation; TM=Text-Messaging

**Table S9. Follow-Up Response by Study Condition – Non-Smartphone Study**

|  | ***Reported Testing* outcome:** | | | | | | | | | | | |
| --- | --- | --- | --- | --- | --- | --- | --- | --- | --- | --- | --- | --- |
|  | 1. **Among all randomized participants** | | | | | | 1. **Among participants who received follow-up messages** | | | | | |
|  | N | n | % | aRR | 95% CI | *P-value* | N | n | % | aRR | 95% CI | *P-value* |
| **Outreach frequency** |  | | | | | |  |  | | | | |
| Every 30 days [Ref] | 15,722 | 1,160 | 7.4% |  |  |  | 14,551 | 1160 | 8.0% |  |  |  |
| Every 10 days | 15,717 | 940 | 6.0% | 0.811 | (0.75, 0.88) | <.0001 | 13,708 | 940 | 6.9% | 0.86 | (0.79, 0.93) | 0.0004 |
| **PN** |  | | | | | |  |  | | | | |
| PN not offered [Ref] | 15,721 | 1,128 | 7.2% |  |  |  | 14,104 | 1128 | 8.0% |  |  |  |
| PN offered | 15,718 | 972 | 6.2% | 0.862 | (0.79, 0.94) | 0.0004 | 14,155 | 972 | 6.9% | 0.859 | (0.79, 0.93) | 0.0003 |

N = total number of participants in the group, n = number of patients who reported their testing outcome, aRR=adjusted risk ratio from the log-binomial model; CI=confidence interval; PN=Patient Navigation; TM=Text-Messaging
